# Time-resolved terahertz–Raman spectroscopy reveals that cations and anions distinctly modify intermolecular interactions of water

**DOI:** 10.1038/s41557-022-00977-2

**Published:** 2022-06-30

**Authors:** Vasileios Balos, Naveen Kumar Kaliannan, Hossam Elgabarty, Martin Wolf, Thomas D. Kühne, Mohsen Sajadi

**Affiliations:** 1 Fritz Haber Institute of the Max-Planck Society, Berlin, Germany; 2 IMDEA Nanociencia, Ciudad Universitaria de Cantoblanco, Madrid, Spain; 3 Dynamics of Condensed Matter and Center for Sustainable Systems Design, Chair of Theoretical Chemistry, University of Paderborn, Paderborn, Germany

**Keywords:** Energy transfer, Raman spectroscopy, Chemical physics

## Abstract

The solvation of ions changes the physical, chemical and thermodynamic properties of water, and the microscopic origin of this behaviour is believed to be ion-induced perturbation of water’s hydrogen-bonding network. Here we provide microscopic insights into this process by monitoring the dissipation of energy in salt solutions using time-resolved terahertz–Raman spectroscopy. We resonantly drive the low-frequency rotational dynamics of water molecules using intense terahertz pulses and probe the Raman response of their intermolecular translational motions. We find that the intermolecular rotational-to-translational energy transfer is enhanced by highly charged cations and is drastically reduced by highly charged anions, scaling with the ion surface charge density and ion concentration. Our molecular dynamics simulations reveal that the water–water hydrogen-bond strength between the first and second solvation shells of cations increases, while it decreases around anions. The opposite effects of cations and anions on the intermolecular interactions of water resemble the effects of ions on the stabilization and denaturation of proteins.

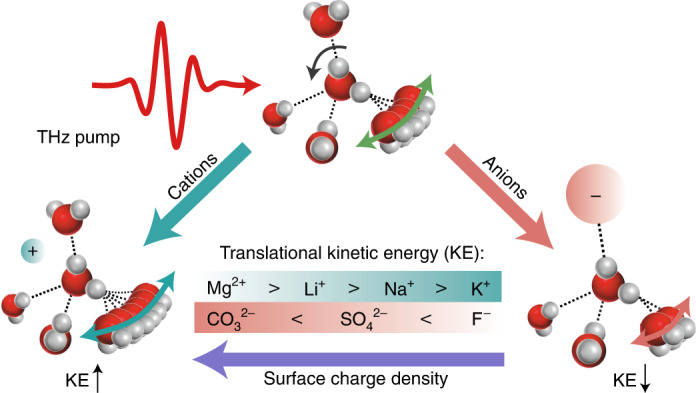

## Abstract (web summary)

Ions in salt solutions perturb the hydrogen bonding between the surrounding water molecules, altering the properties of water, but how ion polarity affects this is not fully understood. By monitoring the dissipation of terahertz energy in salt solutions, it has now been shown that intermolecular rotational-to-translational energy transfer is enhanced by highly charged cations and reduced by highly charged anions.

## Main

Ions are ubiquitous in nature and their solvation is of fundamental importance in chemical and biological reactions^1,2^. They affect the folding and unfolding of proteins and enzymes^3–6^, they are responsible for the transmission of neural signals^7^, they affect chemical equilibria^8^ and they define the efficiency of electrochemical reactions^9–11^. In most if not all of these processes, strong interactions between ions and water molecules are believed to play a central role. Due to electrostatic interactions, ions perturb the dynamics and the local structure of the hydrogen-bonding network of water, although the extent of these effects is not yet fully underestood^12–14^. Ions are typically categorized into structure makers and breakers. Relative to the strength of water–water interactions, the structure makers stabilize the water structure due to their high surface charge density (SCD)^15^ and the structure breakers, consisting of ions with low SCD, interact weakly with water molecules and promote disorder in the hydrogen-bonding network of water^15,16^. However, this SCD-based categorization neglects the polarity of ions. Moreover, the distinct solvation mechanisms of cations and anions are not grounded on a comprehensive, molecular-level description^17,18^.

Extensive theoretical^19–22^ and experimental^23–25^ studies have been conducted to gain microscopic insight into ion solvation. However, due to the complexity of the underlying processes and the selective sensitivity of the employed methods, the pictures that have emerged are still elusive and in many cases contradictory. Some methods are inherently more sensitive to either the effect of anions or that of cations. For instance, ultrafast vibrational spectroscopy, in which the stretch vibration of water’s hydroxyl group is used as a local probe to measure the orientational correlation time of water molecules, is more sensitive to anionic effects^22,26^. Employing this technique, Bakker and co-workers resolved slow hydrogen-bonding dynamics of water molecules that hydrate anions^26^ and found almost no effect on the reorientational dynamics of water molecules surrounding cations^27,28^. In contrast, dielectric relaxation spectroscopy, which probes the dynamics of the molecular permanent dipole moment^29,30^, showed only local ionic effects, limited to the water molecules in the first solvation shell of the cations^31^. On the other hand, neutron scattering resolved the extended distortion of the hydrogen-bonding network and showed structural modifications similar to the impact of pressure on water, but with marginal effect on the hydrogen bonds between water molecules^32^. Interestingly, methods that directly interrogate the hydrogen-bonding network dynamics of water, such as terahertz (THz) and low-frequency Raman spectroscopies, turned out to be triumphant in revealing further details of ion solvation. For the sub-1 THz region, Netz and co-workers theoretically predicted^33,34^ and later experimentally resolved^35^ the ion–water and ion–ion correlation signatures, connecting the ion mobility to the macroscopic electrolyte conductivity. Using high-frequency THz spectroscopy, Havenith and co-workers resolved resonances due to the vibrational motion of ions inside their water cages^36,37^. The isotropic optical Kerr effect spectroscopy by Meech and co-workers resolved a hydrogen-bond vibrational mode, formed between anions and their surrounding water molecules^38,39^. Anisotropic optical Kerr effect spectroscopy by Wynne et al. revealed the jamming of the translational motion of water molecules in strong cationic solutions^29^.

The aforementioned methods measure the ensemble-averaged two-point time-correlation function 〈**A**(0),**A**(*t*)〉, with *t* being time and **A** being the dipole moment vector in THz spectroscopy, the polarizability tensor elements in Raman spectroscopy and the particle density in neutron scattering. More recently, Hamm and co-workers introduced a combined Raman–THz spectroscopy, which measures the three-point cross-correlation of the dipole moment vector **μ** and the polarizability tensor *Π*, that is, 〈*Π*(*t*_1_),**μ**(*t*_2_),**μ**(*t*_3_)〉. They demonstrated the heterogeneity of the local structure of water molecules surrounding cations, and further showed the ability of cations to structure the hydrogen-bonding network of water^18^.

**Fig. 1:**
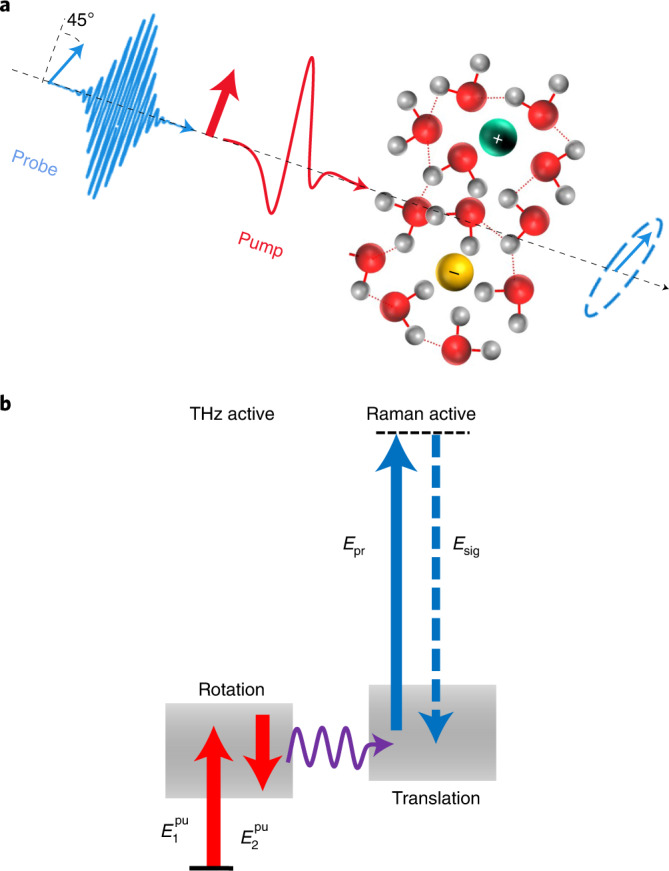
Experimental concept. **a**, An intense THz pump pulse induces optical birefringence in the solution. The effect is monitored by an optical probe pulse that becomes elliptically polarized upon traversing through the medium. **b**, Upon excitation of the rotational degrees of freedom of water with the intense THz field (corresponding to two THz electric-field (*E*) interactions with the system, $E_1^{\rm{pu}}$ and $E_2^{\rm{pu}}$), the deposited energy is rapidly transferred to the translational degrees of freedom and increases its kinetic energy. This causes an increase in the collision rate between molecules, accompanied by the enhancement of the polarizability of the system, which is resolved by a Raman interaction via optical fields *E*_pr_ and *E*_sig_.

Here we employ a similar approach and study the nonlinear THz response of aqueous ionic solutions (Fig. 1a). In contrast to Hamm’s approach, we induce the nonlinear effect with an intense THz pulse via a two-electric-field interaction with the collective permanent dipole moment of the liquid. The response of the system is resolved by an optical–Raman pulse interacting with its collective polarizability. Although the latter field-matter interactions effectively give rise to a two-time-point response function of the form 〈**μ**(0),**μ**(0),*Π*(*t*)〉, the hybrid nature of the interactions provides microscopic insight into the molecular processes^40^. Thus, by selective excitation of an intermolecular mode/process and probing the response of low-frequency Raman-active intermolecular modes of the liquid, we are able to trace in real time the dissipation of the deposited THz energy into the intermolecular degrees of freedom of water^40^.

Using this technique, we recently demonstrated that in liquid water under ambient conditions, a single-cycle pulse centred at ~1 THz resonantly dumps the majority (85%) of its energy into molecular rotations. The deposited energy is transferred quickly (faster than ~300 fs) to the collective translational motions^40^, due to the strong coupling between the intermolecular rotational and translational dynamics in the hydrogen-bonding network of water (Fig. 1b and Supplementary Fig. 1).

In the current study, we use the latter rotational–translational coupling as an intermolecular probe to interrogate the ion-induced perturbations of the hydrogen-bonding network of water. For a systematic study, we select salts that contain a strongly charged cation or anion. The respective counterions are chosen to be almost ‘neutral’; that is, counterion–water and water–water interaction strengths are comparable^41,42^. The cations of choice are potassium (K^+^), sodium (Na^+^), lithium (Li^+^) and magnesium (Mg^2+^) with chloride (Cl^−^) as their counterion. The anions are sulfate (SO_4_^2−^) and carbonate (CO_3_^2−^) with Na^+^ as the counterion, and fluoride (F^−^) with K^+^ as its counterion, for higher solubility. We have also studied the response of MgSO_4_ solutions in which both the cation and the anion are strongly charged. The selected ions have a relatively small electric polarizability, a criterion imposed for reducing potential artefacts emanating from direct contribution of the ion polarizability into the measured signals.

## Results

**Fig. 2:**
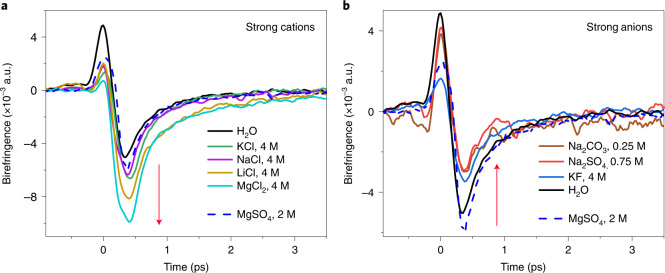
TKE response of aqueous salt solutions. **a**, Terahertz-driven transient optical birefringence of a series of salts with strong cations and Cl^−^ as their counterion, at 4 M concentration. The signal amplitude is cation specific and increases with SCD: MgCl_2_ > LiCl > NaCl > KCl > H_2_O. **b**, The same as **a** but for strong anions at their maximum concentrations with K^+^ or Na^+^ as the counterion. The signal amplitude is anion specific and decreases with SCD: Na_2_CO_3_ < Na_2_SO_4_ < KF < H_2_O. MgSO_4_ salt is composed of a strong cation and a strong anion; hence its birefringence signal at 2 M concentration is added to both panels (dashed blue line). Red arrows indicate the TKE amplitude variation with respect to that of pure water.

Experiments are performed in the transient THz Kerr effect (TKE) configuration by which the THz electric-field-induced optical birefringence is measured (Methods). The resulting TKE signals of all solutions are displayed in Fig. 2 and Supplementary Fig. 4. In both panels the signals are compared with that of pure water. In all signals, two main contributions can be discerned: (1) a coherent instantaneous electronic response and (2) a flipped relaxation tail, which contains information on the nuclear response of the solutions. The amplitude of the latter contribution is highly sensitive to the ionic content of the solutions; it is enhanced by the solutions of chloride salts with strong cations and reduced by solutions of sodium salts (and KF) with strong anions. Moreover, the relaxation tail of all signals can be fit mono-exponentially with decay times in the range ~0.5–0.8 ps (Supplementary Figs. 2, 3 and 5). Additionally, we have measured the THz pump power dependence of the TKE response of pure water and MgCl_2_ solution. As shown in Supplementary Fig. 6, the TKE signals scale with the square of the THz electric field^43^.

**Fig. 3:**
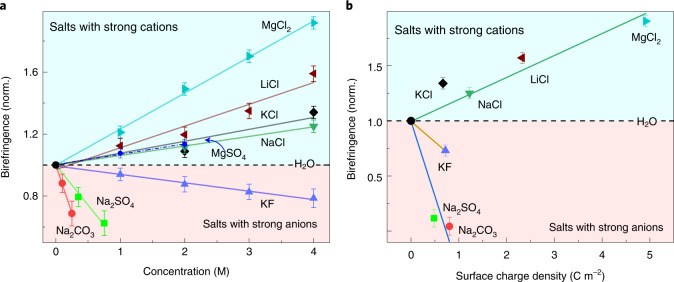
TKE amplitude dependence on concentration and SCD. **a**, The amplitude of the TKE signals, normalized (norm.) to that of pure water, scales linearly with the salt molar concentrations. **b**, The amplitude of the TKE signals relative to that of pure water scales linearly with respect to the gas-phase SCD of the salts. The TKE amplitude of SO_4_^2−^ or CO_3_^2−^ at 4 M is obtained by extrapolating the TKE amplitudes to higher concentrations. As MgSO_4_ bears two highly charged ions, it is not included in **b**. Error bars indicate the standard deviations of the mean determined from the corresponding TKE signals at negative pump–probe delays. The solid lines are linear fits to the data points.

In Fig. 3a,b, we compare the TKE signal amplitudes of all the ionic solutions against the salt concentration and the gas-phase SCD, respectively^44^. Interestingly, for strong cations, the signal amplitudes linearly scale with both the salt concentration and the SCD. For strong anions, a similar trend but with an opposite sign is observed. In general, the slope of the linear fit to the signal amplitudes is steeper for anions than for cations, a possible indication of a stronger anionic versus cationic effect in water. Note also that the TKE signal amplitude of LiCl versus salt concentration exhibits a discernible deviation from linearity (Fig. 3a), likely due to the light mass, high charge density and nuclear quantum effects of Li^+^ (refs. ^45,46^). Similarly, the smallest and lightest anion, namely F^−^, manifests a marked deviation from the other anions in Fig. 3b (refs. ^34,47^).

**Fig. 4:**
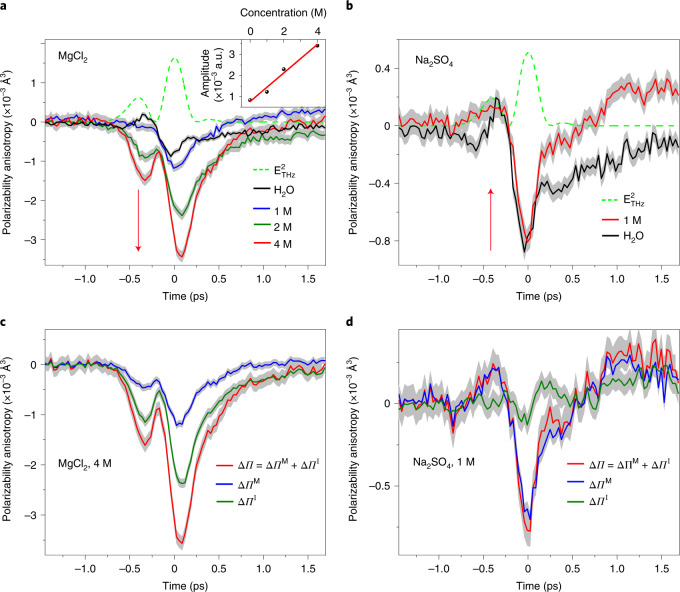
Simulated polarizability anisotropy of the aqueous salt solutions. **a**, Total polarizability anisotropy Δ*Π* of aqueous solutions of MgCl_2_ at 1 M (blue), 2 M (green) and 4 M (red) concentrations. The amplitude increases linearly with concentration, as shown in the inset. **b**, Δ*Π* of Na_2_SO_4_ solution at 1 M (red) exhibits modestly smaller amplitude relative to that of pure water (black). Red arrows in **a** and **b** indicate the increase and decrease of Δ*Π* relative to pure water. $E_{\rm{THz}}^2$, the square of the THz electric field. **c**,**d**, Single molecule Δ*Π*^M^ (blue) and collision-induced Δ*Π*^I^ (green) components of Δ*Π* (red) for MgCl_2_ solution at 4 M and for Na_2_SO_4_ at 1 M, respectively. Notably, while Δ*Π*^I^ is the dominant contribution in the Δ*Π* of the MgCl_2_ solution, its contribution is minor in the Na_2_SO_4_ solution. The shadowed areas indicate standard errors.

The calculated THz electric-field-induced polarizability anisotropies Δ*Π*(*t*) of MgCl_2_ (at 1 M, 2 M and 4 M) and Na_2_SO_4_ (at 1 M) are shown in Fig. 4a,b. The results are obtained from the molecular dynamics (MD) trajectories, using the atomic multipole optimized energetics for biomolecular applications (AMOEBA) polarizable force field and the extended-dipole/induced-dipole (XDID) model as described by Torri^48^, but modified to include the second hyperpolarizability (Methods). Interestingly, relative to pure water, the Δ*Π*(*t*) amplitude of MgCl_2_ is enhanced, while that of Na_2_SO_4_ is reduced. The amplitude drop of the Na_2_SO_4_ solution is modest and not as large as that observed in the experiment, likely due to limitations originating from the employed XDID model (Methods) or the employed force field. The calculated Δ*Π*(*t*) for NaCl (at 2 M and 4 M) replicates the behaviour of MgCl_2_ (Supplementary Fig. 7).

In the following, we show how the changes in the TKE signal amplitude and the corresponding simulated quantity Δ*Π*(*t*) are related to the modification of water–water intermolecular interactions.

## Discussion

In the TKE experiment, due to the action of the pump field polarized along *x* (Fig. 1a), the probe pulse, polarized at 45° relative to *x*, encounters a transient difference Δ*n* = *n*_*x*_ – *n*_*y*_ between the refractive indices along the *x* and *y* directions. The resulting birefringence is given by^43,49^

1 $${\Delta}n \propto {{\langle}\Delta}{\it\varPi}_{xx} - {\Delta}{\it\varPi}_{yy}{\rangle},$$

where Δ*Π*_*ij*_ is the pump-induced change in the collective electronic polarizability tensor element *Π*_*ij*_. Here, *Π* refers to the liquid phase and contains contributions from interactions/collisions between molecules in the condensed phase. The variation Δ*Π* can, in principle, be written as a sum Δ*Π*^M^ + Δ*Π*^I^, whose two contributions arise, respectively, from the intrinsic gas-phase molecular polarizability (*Π*^M^) and the intermolecular interactions and collisions (*Π*^I^) in the condensed phase^50^.

The Δ*Π*^M^ characterizes the degree of anisotropy of the unperturbed *Π* and is usually labelled Δ*α* for single molecules. Averaging Δ*α* over all molecules according to equation (1) yields an expression for single-molecule rotational birefringence Δ*n*_rot_ that scales with the degree of molecular alignment 〈*P*_2_(cos*θ*)〉 = 〈3cos^2^*θ* – 1〉/2, with *θ* being the angle between the molecular dipole vector and the polarization axis of the THz pump pulse, and the molecular polarizability anisotropy Δ*α*, that is, Δ*n*_rot_ ∝ Δ*α*〈*P*_2_(cos*θ*)〉. The averaged Δ*Π*^I^ makes another contribution to the transient birefringence and arises directly or indirectly from the pump-induced changes in the collision-induced polarizability. Additionally, THz electric-field-induced ionization and charge separation may contribute to the total polarizability of water^51,52^. However, as demonstrated by Elsässer and co-workers, the latter contributions modulate the isotropic part of the complex dielectric response of water, with negligible impact on its anisotropic response, measured in the TKE experiment^51,52^.

As summarized schematically in Fig. 1b, the THz energy deposited into the rotational degrees of freedom of water is transferred into the translational motion of the neighbouring water molecules^40,53^. In a previous paper^40^, we showed that the decay of the TKE signal’s tail reveals the relaxation of the translational motion of water molecules, most likely that of the hydrogen-bond bending mode due to its spectral proximity to the excitation frequency (Supplementary Figs. 1–5). Therefore, given the initial excitation of the rotational dynamics of water with the THz pump pulse, the rotation-to-translation energy transfer causes a coherent increase in the rate of the collisions between water molecules, which can be resolved as a change in the refractive index of the liquid via Δ*Π*^I^.

In light of the TKE response of pure water, the ion-induced enhancement/weakening of the TKE signals may indicate underlying changes in the strength of the intermolecular water–water hydrogen-bonding interactions, by which the rotational–translational coupling is modified. To examine the soundness of this proposition, we first decompose Δ*Π* into the contributions ﻿Δ*Π*^M^ and Δ*Π*^I^. The corresponding results for MgCl_2_ at 4 M, NaCl at 4 M and Na_2_SO_4_ at 1 M are shown in Fig. 4 and Supplementary Fig. 7. Remarkably, while in MgCl_2_ and NaCl solutions the collision-induced polarizability anisotropy Δ*Π*^I^ makes the dominant contribution to the total polarizability anisotropy, its contribution in the Na_2_SO_4_ solution is minor.

**Fig. 5:**
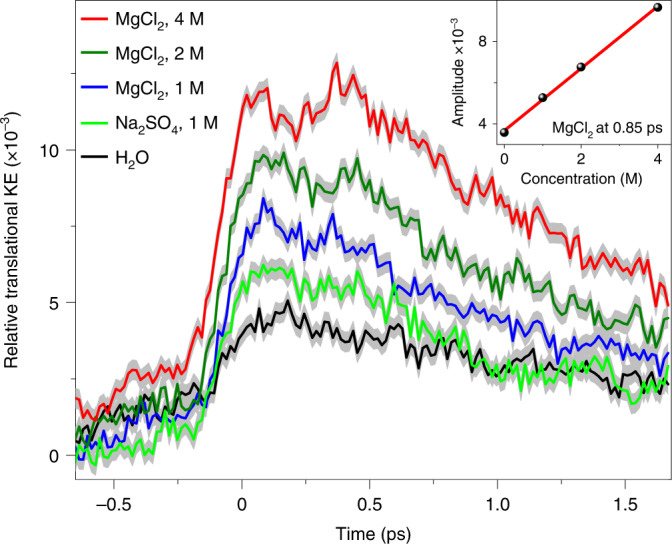
Calculated THz-induced perturbation of molecular KE distribution. Temporal evolution of the ratio of the molecular translational KE to the total instantaneous KE of aqueous ionic solutions of MgCl_2_ at 1 M (blue), 2 M (dark green) and 4 M (red), Na_2_SO_4_ at 1 M (light green) and pure water (black), obtained from polarizable force field MD (pFFMD) simulations. The deviation of the ratio from the equilibrium value of 1/3 is plotted, so that a positive value indicates a relative increase in the respective KE contribution in comparison to an equilibrium (equipartitioned) distribution. Note that for the strong cation Mg^2+^, the amplitude of the perturbation of the KE distribution substantially increases in comparison to the strong anion, SO_4_^2−^. Inset: the linear concentration dependence of the relative increase in translational KE (taken at *t* = 0.85 ps) in MgCl_2_ solutions. The shadowed areas indicate standard errors.

Along the same line, the increase/decrease of the collision-induced polarizability may also be observed in the kinetic energy (KE) of the translational motion of water molecules. Thereby, we calculate the transient excess translational KE(*t*) of water molecules in MgCl_2_ and Na_2_SO_4_ solutions. As shown in Fig. 5, the translational KE(*t*) of water molecules in MgCl_2_ solutions is substantially enhanced relative to pure water. As displayed in the inset of the same figure, the enhancement scales linearly with the salt concentration. The KE(*t*) of the water molecules in NaCl solutions also shows a similar trend (Supplementary Fig. 8). For the Na_2_SO_4_ solution, although the expected decrease in the translational KE(*t*) is not observed, its amplitude is not as large as that of the MgCl_2_ solution at the same concentration. This parallels our previous observation that the employed parameterization of the polarizable force field underestimates the effects of Na_2_SO_4_ in the polarizability calculations.

To connect the latter findings with the local intermolecular interactions in water, we resort to equilibrium ab initio MD (AIMD) simulations and calculate the water–water hydrogen-bond strength. For quantifying the strength of the hydrogen bonds, we employ energy decomposition analysis for condensed phase systems based on absolutely localized molecular orbitals (ALMO-EDA)^54,55^ within Kohn–Sham density functional theory (Methods). The hydrogen bonds are categorized into four disjoint classes: those between the first and second solvation shells of an anion; the same for cations; those involving one water molecule, which is simultaneously shared in the first solvation shell of an anion and a cation; and those between the remaining bulk-type water molecules, meaning that these hydrogen bonds are between two water molecules, both of which are not in the first solvation shell of an ion.

**Fig. 6:**
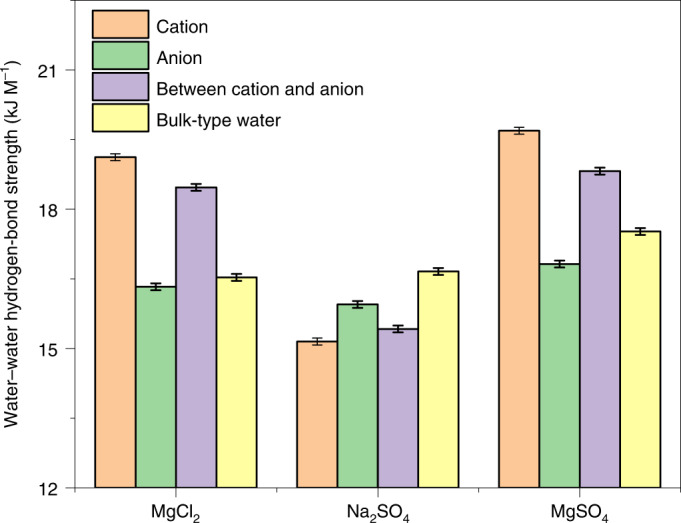
Water–water hydrogen-bond strength in aqueous salt solutions. Hydrogen-bond strength from equilibrium trajectories of AIMD simulations are obtained for every water molecule in the system, classified into four disjoint categories: from left to right, a water molecule in the solvation shell of a cation (orange), an anion (green), a water molecule shared between a cation and an anion (violet) and the remaining bulk-type molecules (yellow). For water molecules in the first solvation shell of an ion, the hydrogen bonds correspond to those between water molecules in the first and second solvation shells of that ion. Notably, while the water–water hydrogen-bond strength between the first and second solvation shells of cations increases, it decreases around anions. We have used a simple geometric definition of a hydrogen bond, with an O–O distance of <3.5 Å and an angle of <30°. Error bars indicate standard errors.

As shown in Fig. 6, by comparing the water–water hydrogen-bond strength around ions with that of the bulk-type water, the following conclusions can be drawn: In MgCl_2_ solution, the Mg^2+^ ions with their high charge density lead the water–water hydrogen bonds in their immediate surroundings to be 2–3 kJ mol^–1^ stronger than more distant hydrogen bonds. This is in line with a recent experimental study by Shalit et al. denoting the ability of strong cations such as Mg^2+^ to structure the hydrogen-bonding network of water^18,56^. On the other hand, in the Na_2_SO_4_ solution, the SO_4_^2−^ ions show an opposite trend, namely the neighbouring hydrogen bonds are slightly weaker. Along the same lines, by analysing the changes in the OH-stretch vibration frequency of water molecules in the first and second solvation shells of CO_3_^2−^, Yadav et al. reported the weakening of the water–water hydrogen-bonding strength around CO_3_^2−^ (ref. ^57^). Previous ab initio calculations on solvated clusters have also shown this increase (decrease) of the hydrogen-bond strength between the first and second solvation shells of cations (anions)^58^.

Thereby, the emerging picture thus far is as follows. Ions influence the water–water intermolecular hydrogen-bond strength between the first and second solvation shells. Highly charged cations strengthen this interaction and highly charged anions weaken it. The ion-induced change in the water–water interaction strength alters the intermolecular coupling between the rotational and translational degrees of freedom. As such, the THz energy initially deposited into the intermolecular rotational motion of water funnels into the translational motions, with the efficiency determined by the ionic content of the solution, and influences the collision-induced contribution to polarizability anisotropy, which is captured in our TKE experiment.

We now consider the THz response of MgSO_4_, a system in which both the anion and the cation are strongly charged, to investigate whether the impact of two strong ions coexisting in a solution is additive or cooperative. As shown in Figs. 2 and 3a and Supplementary Fig. 4, the TKE signal amplitude of MgSO_4_ relative to the signal amplitudes of MgCl_2_ and Na_2_SO_4_ displays an intermediate behaviour: in contrast to Na_2_SO_4_, the TKE signal of MgSO_4_ is larger than the signal of pure water, while it is smaller than that of MgCl_2_. Moreover, as shown in Fig. 6, the water–water hydrogen-bond strength around Mg^2+^ and SO_4_^2−^ in MgSO_4_ solution differs from that in MgCl_2_ and Na_2_SO_4_ solutions. For MgSO_4_, the average of all ion-mediated contributions is larger than the bulk-type water–water hydrogen-bond strength, signifying its ‘cationic character’. Notably, this indicates a non-additive but cooperative effect of ions in MgSO_4_ solution^26,59^, since in solutions with ‘neutral’ counterions, SO_4_^2−^ shows a larger effect compared to Mg^2+^, as seen in Fig. 3a.

In light of these results, the linear concentration dependence of the TKE signal amplitude in strong cationic solutions, holding even at elevated concentrations, may now be understood. In particular, in the extreme case of the MgCl_2_ solution at 4 M, all water molecules are consumed in the first solvation shells of ions; hence, there is virtually no free water to form a second hydration shell for each ion^32^. Nevertheless, we still observe a linear trend in the concentration dependence of the experimental, as well as the calculated, quantities (Fig. 3a and insets in Figs. 4a and 5). We believe that in such solutions, the role of Cl^−^ in water–water interactions is critical. The water–Cl^−^ interaction strength is comparable with that of water–water in bulk (Fig. 6), and the dynamics of water molecules solvating Cl^−^ are minimally perturbed by Cl^−^ (ref. ^60^). Thereby, there are still abundant water molecules in the system whose dynamics and hydrogen-bond strengths resemble that of bulk, by which the second solvation of Mg^+2^ at high concentrations can be formed.

To summarize, our joint TKE experiment and MD simulations in a large set of aqueous ionic solutions revealed that strong cations enhance the total polarizability anisotropy of water, whereas strong anions weaken this effect, relative to that in pure water. The polarizable classical MD simulations successfully reproduce the experimental results and further show that the collision/interaction-induced polarizability is enhanced in the presence of strong cations and weakened by strong anions. The origin of these effects is attributed by AIMD simulations to the changes in the water–water hydrogen-bonding interaction between the first and second solvation shells of ions; strong cations make hydrogen bonds stronger, while strong anions weaken them.

Notably, although ions with high SCD, irrespective of their polarity, interact strongly with their immediate surrounding water molecules and structure water in their first solvation shells^61–65^, the hydrogen-bond strength between the first and second solvation shells depends not only on the SCD but also on the polarity of the ions. This subtle difference distinguishes our findings from the traditional SCD-based categorization of ions into structure makers and breakers, as discussed in the introduction.

Finally, the opposite impact of strong anions and cations on the water–water intermolecular interactions is in line with the impact of ion polarity on the stabilization and denaturation of proteins’ tertiary structure in aqueous solutions (that is, the Hofmeister effect)^66,67^. While strongly hydrated cations denature proteins, mainly by direct preferential binding to the polar amide groups or by pairing with the protein’s negatively charged carboxylate groups^5,68,69^, the mechanism of protein stabilization by strongly hydrated anions remains elusive. Our findings regarding the weakening of the water–water hydrogen-bond strength in the presence of strong anions may support the mechanism suggested by Collins et al., according to which the anion-induced protein stabilization is water mediated and attributed to the ‘interfacial effects of strongly hydrated anions near the surface of proteins’^70^. Less efficient energy transfer in water and weaker water–water intermolecular interactions may result in converting the liquid into a ‘less good solvent’ by strong anions, which may drive the proteins to minimize their solvent-accessible surface area by folding^70^.

## Methods

### Experiment

The THz–Raman experiment is performed in the TKE configuration, whose details are given elsewhere^43,71,72^. As shown schematically in Fig. 1a, a linearly polarized THz electric field with a strength of ~2 MV cm^–1^ pumps the aqueous salt solutions. The liquid samples (thickness of 100 µm) are held between a rear glass window and a 150-nm-thick silicon nitride (SiN) membrane as the entrance window^73^. The SiN thin window exhibits a negligible Kerr signal and, therefore, eases the challenges for separating the liquid response from that of the window^73^. The induced transient birefringence, Δ*n*(*t*), is measured by a temporally delayed and collinearly propagating probe pulse (800 nm, 2 nJ, 8 fs), whose incident linear polarization is set to an angle of 45° relative to the THz electric-field polarization. Due to the pump-induced birefringence, the probe field components polarized parallel (∥) and perpendicular (⟂) to the pump field acquire a phase difference Δ*ϕ* when propagating through the sample, thereby resulting in elliptical polarization. The Δ*ϕ* is detected with a combination of a quarter-wave plate and a Wollaston prism, which split the incoming beam in two perpendicularly polarized beams with power *P*_∥_ and *P*_⟂_. In the limit |Δ*ϕ*| ≪ 1, the normalized difference *P*_∥_ – *P*_⟂_ fulfills $\frac{{P_\parallel - P_ \bot }}{{P_\parallel + P_ \bot }} \approx {\Delta}\phi$. The measured phase shift is related to the change in the refractive index Δ*n* of the liquid, that is, Δ*ϕ* = Δ*nLωc*^–1^, where *L* is the thickness of the sample, *ω* is the probe frequency and *c* is the speed of light.

### Samples

All samples were prepared volumetrically by adding Milli-Q water to the weighted amount of salt. The salts were all handled in a glove box to avoid further water uptake during the preparation and gain maximum control of the water concentration in each solution. All solutions were prepared up to 4 M with a few intermediate concentrations. For salts with lower solubility, that is, Na_2_SO_4_ and Na_2_CO_3_, we reached close to the solubility limit and prepared an additional intermediate concentration. For fluoride (F^−^), we chose potassium (K^+^) as its counterion instead of sodium (Na^+^), due to its higher solubility.

### MD simulations

In aqueous ionic solutions, a combination of very strong ionic electric fields and field gradients; long-range interactions; and the interplay of polarization and charge transfer effects make the simulations a challenging feat^74^. For instance, ignoring the charge transfer polarization effects, as is done in simple point-charge models, can lead to the erroneous conclusion that all ions are structure makers^75,76^. Even for a simple electrolyte solution such as NaCl, the force fields based on point charges were found unable to reproduce the concentration dependence of important thermodynamic properties under ambient conditions^77^. For the current study, we found that a force field based on a simple point-charge model (the SPC force field) fails to reproduce any concentration dependence of the THz signal (Supplementary Fig. 9).

Given the substantial amount of statistical sampling that is required to obtain the THz pulse-induced polarizability anisotropy, we have resorted to non-equilibrium pFFMD simulations using the AMOEBA force field^78^. We have performed pFFMD simulations on the ionic solutions MgCl_2_, NaCl and Na_2_SO_4_. Compared to simple point-charge models, AMOEBA assigns to each atom a permanent partial charge, a dipole and a quadrupole moment. Electronic many-body effects are also represented using a self-consistent dipole polarization procedure. Due to the immense electric fields of ions, this explicit treatment of polarization becomes important in order to reproduce the THz Kerr response and its concentration dependence (Supplementary Fig. 9).

In our non-equilibrium MD simulations, the external field is directly included in the Hamiltonian that is used to time-propagate the system. The essential idea is that for a system perturbed from equilibrium, all non-equilibrium properties can be computed using equilibrium averaging of dynamical properties, time evaluated under the full (perturbed) dynamics^79,80^. This is in contrast to an equilibrium MD-based approach, where under a linear response assumption, the response is computed as an ensemble-averaged time-correlation function. The direct non-equilibrium approach offers two advantages: First, by simulating a non-equilibrium system, one can visualize microscopically the physical mechanisms that are important to excitation and relaxation, including the distortions of the local molecular structure, the processes of molecular alignment and rotation, and transport processes like the aforementioned transient energy fluxes. Second, the validity of the approach is not limited to the linear response regime. The downside is that one needs perturbation strengths that are huge in order to produce a detectable response that is larger than the statistical noise, but this is becoming less important with the current intensities of laser pulses. Nevertheless, under the employed electric-field strength, obtaining an acceptable signal-to-noise ratio required the simulation of 25,000 trajectories for each investigated system.

The analysis of hydrogen-bond strengths is based on equilibrium AIMD simulations of the same systems used in the pFFMD. In AIMD, the electronic structure, which is optimized on the fly at each MD time step, responds adiabatically to the instantaneous local fields, so that—unlike in polarizable force fields—there is no imposed artificial partitioning of the electronic density into separate non-overlapping entities. Charge transfer and polarization effects are naturally accounted for, and finite-size effects are also fully included, so that the strength of the electric field varies at each point of space even on submolecular distances. AIMD simulations of aqueous ionic solutions have been found to give reliable results, provided that dispersion effects are carefully treated^74^.

### KE decomposition

In the pFFMD simulations, at each MD snapshot, the translational KE of each molecule is computed from the velocity of the molecular centre of mass. The rotational KE is then calculated as the difference between the total molecular KE and the KE of the molecular centre of mass.

### Hydrogen-bond ALMO-EDA analysis

For quantifying the strength of the hydrogen bonds, we employ ALMO-EDA^54,55^ within Kohn–Sham density functional theory. Conceptually, ALMO-EDA decomposes intermolecular interaction energies first by filtering out the frozen electrostatic and polarization effects from the total many-body intermolecular binding energy. The remaining charge transfer contribution is then split into pairwise two-body terms, each corresponding to an individual hydrogen bond or ion–water interaction in the system. These two-body terms are obtained self-consistently under fully periodic boundary conditions. The water–water hydrogen-bond strength is then calculated for all pairs of molecules.

### pFFMD

We performed pFFMD simulations on three different aqueous electrolyte solutions—MgCl_2_ (1 M, 2 M and 4 M), NaCl (2 M and 4 M) and Na_2_SO_4_ (1 M)—plus on pure liquid water (as a reference). Supplementary Table 1 lists the details of the simulated systems. Simulations were performed under fully periodic boundary conditions and a time step of 0.4 fs. For each system, we generated 25,000 non-equilibrium MD trajectories. Initial configurations were uniformly sampled from equilibrated canonical ensemble MD simulations. For each configuration, the thermostat was switched off (microcanonical ensemble) and then a THz electric-field pulse, identical to the one used in our experiment, was applied in the *x* direction after 0.1 ps. To improve the signal-to-noise ratio, we used a pulse amplitude that is eight times stronger than the experimental one.

Simulations were performed using the Tinker software^81^, using double precision floating point numbers. The velocity Verlet algorithm was employed to time-propagate the positions and velocities of atoms. The AMOEBA force field parameter set ‘amoebanuc17’ was used^78,82–84^. For the sulfate ion, the parameters were taken from ref. ^85^. A buffered 14-7 potential^86^ was used to include the effects of a short-range repulsion–dispersion interaction. Short-range interactions were truncated at 9 Å, whereas the smooth particle mesh Ewald^87^ was employed to treat the long-range electrostatic interactions. The mutual induced dipoles were computed using the conjugate gradients method^88,89^ with a tolerance of 0.00001 debye. For inclusion of the external electric field *E* in energy and force calculations in Tinker, we introduced into the code the electric force term $F = k_1qE$ and induced-dipole term $\mu = k_2\alpha E$, where *q* is the atomic charge. The constants *k*_1_ = 1,185.85 and *k*_2_ = 3.567 give the forces and dipoles in kilocalories per angstrom per mole and square angstroms, when the electric field *E* and polarizability *α* are in atomic units and cubic angstroms. The existing implementation of conjugate gradient minimization in Tinker was utilized to compute the mutual induced dipoles that produce the local field at each molecule.

The time-dependent polarizability anisotropy results reported in this work were averaged over all the 25,000 trajectories. For all our analyses involving hydrogen bonds, we have used a standard geometric definition of the hydrogen bond (O–O distance of <3.5 Å and hydrogen-bond angle of <30°)^90^.

### AIMD

To study the hydrogen-bond strength between water molecules in ionic solutions, we performed AIMD simulations under field-free conditions for pure liquid water (as reference) and for MgCl_2_ (2 M), Na_2_SO_4_ (1 M) and MgSO_4_ (2 M). For each system, five independent MD trajectories were simulated. Each trajectory was first equilibrated for 10 ps in the canonical ensemble, followed by a 50 ps production run under the microcanonical ensemble. The AIMD simulations were conducted using the Quickstep module of the CP2K software^91^. Throughout, the energies and forces were computed using the mixed Gaussian/plane-waves approach^92^, with the Kohn–Sham orbitals represented by an accurate triple-zeta basis set with two sets of polarization functions (TZV2P)^93^. Plane waves with a cut-off of 400 Ry were used to represent the charge density, whereas the core electrons were described by the Goedecker–Teter–Hutter pseudopotentials^94,95^. The BLYP exchange–correlation functional was used together with a damped interatomic potential for dispersion interactions (Grimme-D3)^96^. Simulations were conducted using a time step of 0.4 fs.

The average hydrogen-bond strengths shown in this work were obtained by performing ALMO-EDA for 500 AIMD snapshots uniformly sampled from the 50 ps production trajectories. The technical details behind ALMO are described elsewhere^55^. All ALMO-EDA calculations in this work were performed using the ALMO-EDA implementation in the CP2K program^91^ with the same settings as described in our AIMD simulations.

### Calculation of polarizability anisotropy

The polarizability anisotropy was calculated from the ensemble-averaged difference between the *xx* component of the polarizability tensor, and the average of the remaining two diagonal components, *yy* and *zz* (laboratory reference frame, with the *x* axis being defined by the THz electric field):

$$\Delta \alpha = \alpha _{xx} - \left( {\frac{{\alpha _{yy} + \alpha _{zz}}}{2}} \right).$$

The total polarizability of each component can be calculated by summing the permanent (*α*^perm^) and induced (*α*^ind^) polarizabilites of all the molecules in the system:

$$\alpha _{\mathrm{total}} = \mathop {\sum }\limits_{i = 1}^n \left( {\alpha _i^{\mathrm{perm}} + \alpha _i^{\mathrm{ind}}} \right)$$

where *n* is the total number of molecules.

The induced polarizability of each molecule was computed using an XDID mechanism in a self-consistent field manner^48^. The model is ‘extended’ by the inclusion of both the first and second hyperpolarizabilities. The self-consistent field equation for the first and second hyperpolarizability XDID mechanisms is given by the following expression:

$${\alpha _i^{\mathrm{ind}} = \alpha _i^{\mathrm{perm}}\mathop {\sum }\limits_{j\neq i}^n T_{ij}\left( {\alpha _j^{\mathrm{perm}} + \alpha _j^{\mathrm{ind}}} \right) + \beta _i\bf{ {E}}{_i} + \frac{{\gamma _i\bf{{E}}{_i}} ^2}{2}}$$

where ${T_{ij} = 3 {{\bf{r}}_{\bf{ij}}}\times {r_{ij}} /r_{ij}^5 - I/r_{ij}^3}$ is the standard dipole–dipole interaction tensor between molecules *i* and *j*; **r**_*ij*_ and *r*_*ij*_ are the distance vector and norm distance from molecule *i* to *j*, respectively; *I* is a 3 × 3 unit tensor; and *β*_*i*_ and *γ*_*i*_ are the first and second hyperpolarizability tensors of molecule *i*, respectively, which are calculated in gas-phase conditions using our parameters in Supplementary Table 2. The **E**_*i*_ is the local electric-field vector at molecule *i* created only by all the surrounding molecules (that is, the permanent ionic charges and the permanent and induced dipoles of all entities but excluding the external terahertz field) within a 7.5 Å distance from the centre of mass of the molecule. We have verified the validity of this cut-off by replicating the simulation box in all directions and increasing the cut-off distance to 15 Å, which gave only a negligible change in anisotropy. The self-consistent field equation was solved iteratively until the solution reached the convergence tolerance of 0.000001 Å^3^.

The induced dipole moment of each molecule was obtained using the preconditioned conjugate gradient method^88,89^ with a convergence tolerance of 0.000001 debye and with the following initial guess, residue and direction:

$$\begin{array}{lll}{\mathrm{Initial}}\ {\mathrm{guess}}:{\mathbf{\upmu}}{_i^{{\mathrm{ind}},0}} = {\alpha }_i^{{\mathrm{perm}}} \\ \left[ {\mathbf{E}}_{{\mathrm{external}}}+\mathop {\sum }\limits_{j\neq i}^n \frac{q\cdot {\hat{\mathbf{r}}}_{ij}}{{r_{ij}^2}} - \mathop {\sum }\limits_{j\neq i}^n \frac{1}{{r_{ij}^3}}\left({\mathbf{\upmu}}_j ^{{\mathrm{perm}}} \right)+ \mathop {\sum }\limits_{j\neq i}^n \frac{3}{{r_{ij}^5}} \left( {\mathbf{r}}{_{ij}}\cdot\left({\mathbf{\upmu}}_j ^{{\mathrm{perm}}} \right) \right)\cdot{{\mathbf {r}}_{ij}} \right] \end{array}$$

$${{\mathrm{Initial }}\ {\mathrm{residue}}}:{\bf{r}}_i^0 = \mathop {\sum }\limits_{j\neq i}^n T_{ij}\left( {\mathbf{\upmu}_j^{{\mathrm{ind}},0}} \right)$$

$${{\mathrm{Initial }}\ {\mathrm{direction}}}: {\bf{p}}_i^0 = {\alpha} _i^{{\mathrm{perm}}}{\bf{r}}_i^0$$

where ${\hat{\bf{r}}}_{\bf{ij}}$﻿ is the unit vector pointing from molecule *i* to *j*, *q* is the molecular charge and **E**_external_﻿ is the externally applied field vector.

### Parametrization of the dipole moments, polarizabilities and hyperpolarizabilities

The permanent dipole moment of the ions was set to zero, while for water a gas-phase dipole moment of 1.93 debye was assigned along the molecular bisector. The ionic polarizability parameters from refs. ^97,98^ were used to model the permanent polarizabilites of the ions, whereas the polarizability tensor of water was calculated in the gas phase. All the employed parameters are listed in Supplementary Table 2. The values of the dipole moment and polarizability parameters of gas-phase water reported in this work were parameterized using the CP2K program^91^, while the first and second hyperpolarizability parameters of gas-phase water were computed using the DALTON program^99^. For parameterization we used the same settings as described in our AIMD simulations.

## Online content

Any methods, additional references, Nature Research reporting summaries, source data, extended data, supplementary information, acknowledgements, peer review information; details of author contributions and competing interests; and statements of data and code availability are available at 10.1038/s41557-022-00977-2.

## Supplementary information

Supplementary Information Supplementary Figs. 1–9 and Tables 1 and 2.

## Data Availability

The raw data underlying all the figures, as well as the full data needed to support, interpret, verify and extend the research in the article, can be downloaded at 10.5281/zenodo.6514905.

## Code availability

The source codes needed to support, interpret, verify and extend the research in the article can be downloaded at 10.5281/zenodo.6514905. The source code for the calculation of the polarizability anisotropy is available at https://github.com/NaveenKaliannan/Dipole-Polarisability.
