# Supplementary material for: Time-resolved terahertz–Raman spectroscopy reveals that cations and anions distinctly modify intermolecular interactions of water
Source: Nat Chem. 2022 Jun 30;14(9):1031–7. doi: 10.1038/s41557-022-00977-2 (PMC9417992; doi:10.1038/s41557-022-00977-2)
Supplement: Supplementary file 1 — Supplementary Figs. 1–9 and Tables 1 and 2. [file 41557_2022_977_MOESM1_ESM.pdf]

nature portfolio

https://doi.org/10.1038/s41557-022-00977-2

Supplementary information

Time-resolved terahertz–Raman spectroscopy reveals that cations and anions distinctly modify intermolecular interactions of water

In the format provided by the authors and unedited

Time resolved THz-Raman spectroscopy reveals that cations and anions distinctly modify intermolecular interactions of water

Vasileios Balos[1,3*], Naveen Kumar Kaliannan[2], Hossam Elgabarty[2,*], Martin Wolf[1], Thomas D. Kühne[2], Mohsen Sajadi[1,2,*]

[1]*Fritz Haber Institute of the Max-Planck Society, Berlin, Germany*

[2]*Dynamics of Condensed Matter and Center for Sustainable Systems Design, Chair of Theoretical Chemistry, University of Paderborn, Paderborn, Germany*

[3]*IMDEA Nanociencia C/Faraday 9 Ciudad Universitaria de Cantoblanco, Madrid, Spain*

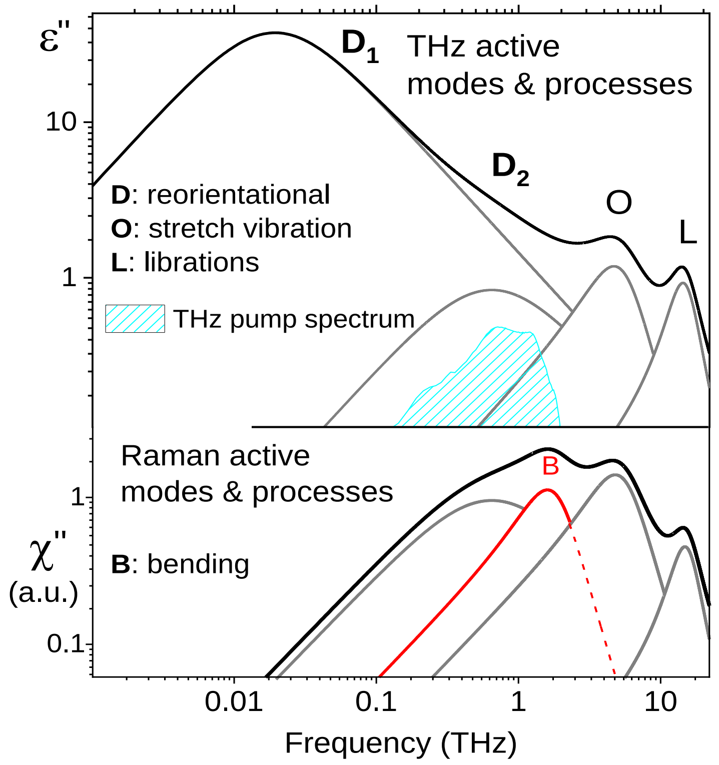

Supplementary Fig. 1. Equilibrium dielectric loss ($\varepsilon''(\omega)$) and incoherent Raman spectra of water.[1] Two Debye processes and two vibrations (network stretch vibrations and single-molecule hindered rotation, libration) are typically sufficient to fit the dielectric spectrum of water.[1] The Raman spectrum of water lacks the first Debye process,[1] but the H-bond bending vibration (red line) gains a significant amplitude. The spectra of the excitation THz fields at ~1 is indicated by the cyan dashed areas. a.u., arbitrary units. Adapted (Fig. 2) with permission from Fukasawa et al., PRL, 95, 197802, 2005 (https://doi.org/10.1103/PhysRevLett.95.197802). Copyright (2005) by American Physical Society.

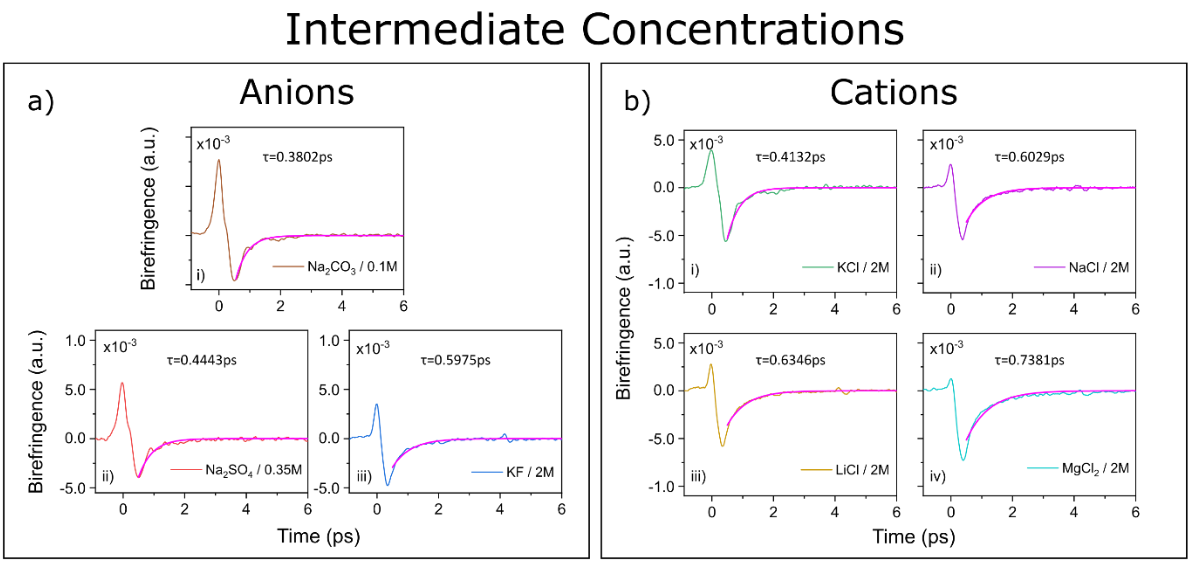

Supplementary Fig. 2. Transient optical birefringence of intermediate salt concentrations of a) strong anions and b) strong cations. The tail (above 0.5ps) is fitted with a single exponential function and the resulting fitted curve is shown in each figure (magenta line). The time constants returned from the fitting routine are mentioned in each figure. Note that each time trace has been normalized to the value of the corresponding water amplitude in the day of measurement, to avoid errors from the THz power fluctuations.

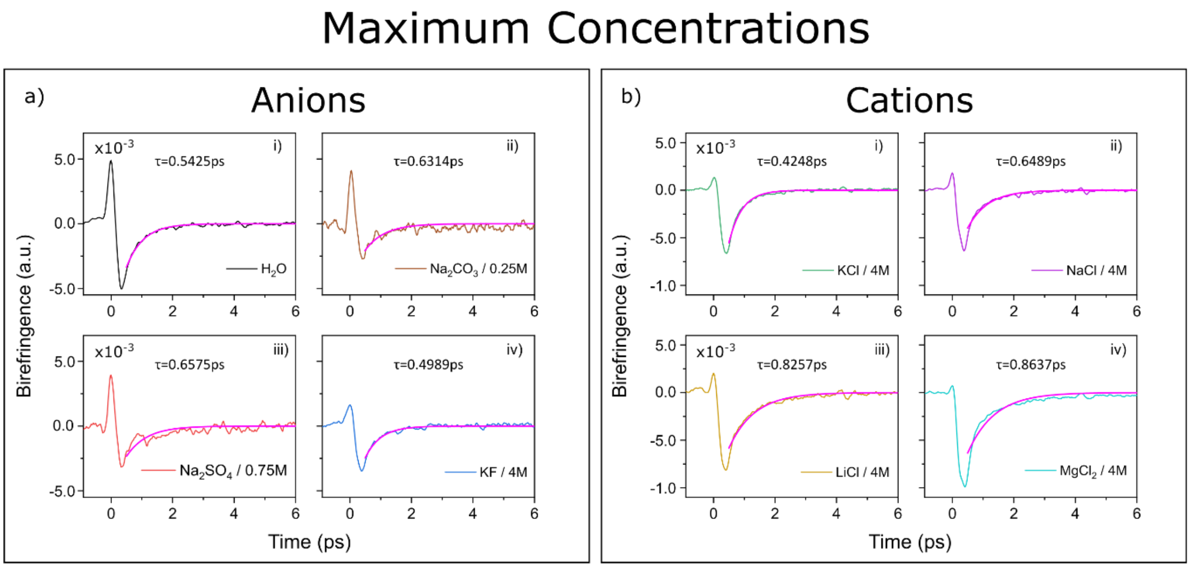

Supplementary Fig. 3. Transient optical birefringence of maximum salt concentrations of a) strong anions and b) strong cations. The tail (above 0.5ps) is fitted with a single exponential function and the resulting fitted curve is shown in each figure (magenta line). The time constants returned from the fitting routine are mentioned in each figure. Note that each time trace has been normalized to the value of the corresponding water amplitude in the day of measurement, to avoid errors from the THz power fluctuations.

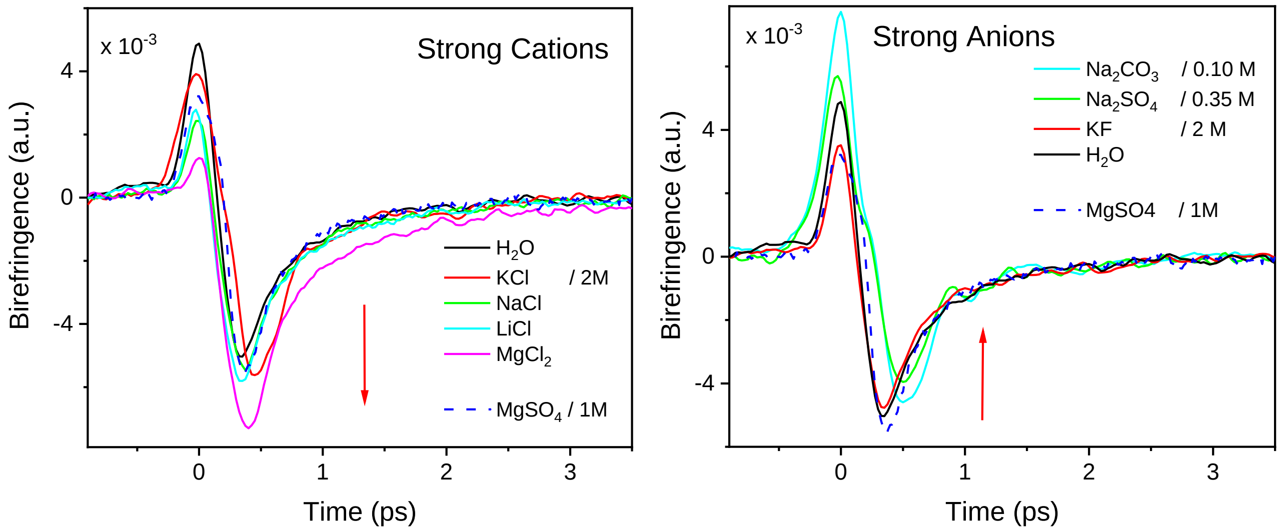

Supplementary Figure 4. Transient optical birefringence of intermediate concentrations (values stated at the legend) of: a) strong cations: KCl (red line), NaCl (green line), LiCl (cyan line), $MgCl_2$ (magenta line) and b) strong anions: KF (red line), Na_2SO_4 (green line), Na_2CO_3 (cyan line), compared to pure liquid water (black line). The amplitude of the TKE signal drastically changes in the presence of highly charged electrolytes and the change is ion specific. The red arrows indicate the increase in the SCD of the ions. The blue dashed lines indicate the response of 1M of $MgSO_4$ solution.

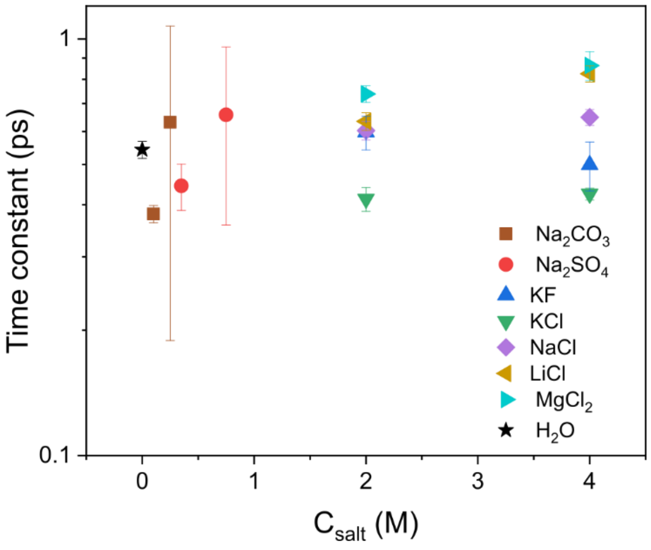

Supplementary Fig. 5. Time constant values of Na_2CO_3 (brown squares), Na_2SO_4 (red circles), KF (blue up-triangles), KCl (green down-triangles), NaCl (purple diamond), LiCl (orange left-triangles), $MgCl_2$ (cyan right-triangles) and H_2O (black star), returned from the fitting of the tail (above 0.5ps) of the TKE signal with a single exponential function, versus salt concentration. Error bars correspond to the individual coefficient of determination (R^2) of each fit.

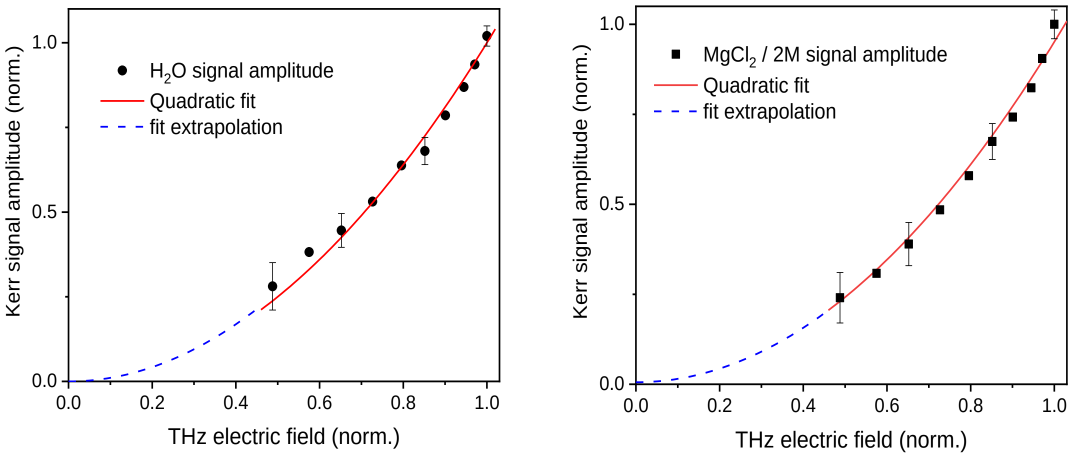

Supplementary Fig. 6. Fluence dependence of the TKE response of water (left) and 2 M solution of $MgCl_2$ shows that both the TKE signals scale quadratically with THz electric field. The red solid line indicates the quadratic fit, while the blue dashed line its extrapolation to lower E-field intensities.

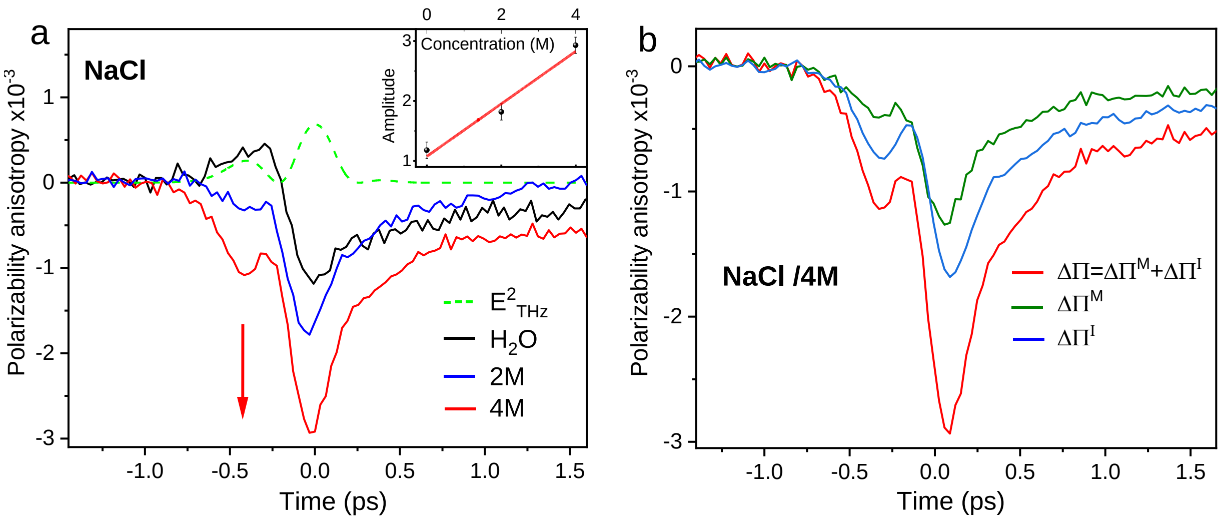

Supplementary Fig. 7. a, Calculated total polarizability anisotropy $\Delta\Pi$ in water (black solid line) and aqueous solutions of NaCl at 2M (blue solid line) and 4M (red solid line) concentrations. The arrow indicates the increase of the $\Delta\Pi$ amplitude relative to pure water. The green dashed line indicates the square of the THz E-field (E^2_{THz}). The inset shows the amplitude of the signals versus salt concentration. **b**, $\Delta\Pi$ (red line) is decomposed into single molecule $\Delta\Pi^M$ (green line) and collision induced $\Delta\Pi^I$ (blue line) polarizability component for NaCl solution at 4M.

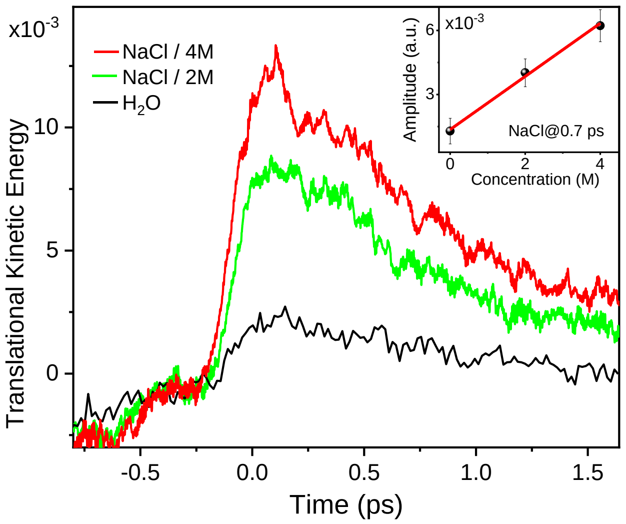

Supplementary Fig. 8. Calculated temporal evolution of the ratio of the molecular translational KE to the total instantaneous KE of water (black line) and aqueous NaCl solutions at 2M (green line) and 4M (red line), obtained from polarizable FFMD simulations. The deviation of the ratio from the equilibrium value of one-third is plotted so that a positive value indicates a relative increase in the respective KE contribution in comparison to an equilibrium (equipartitioned) distribution. The inset shows the amplitude of the signals versus salt concentration.

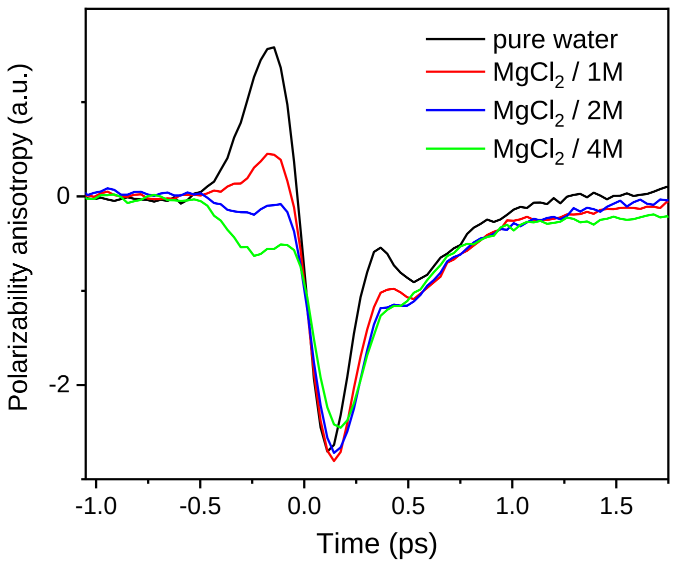

Supplementary Fig. 9. Calculated transient optical birefringence of pure water (black line) and $MgCl_2$ solutions at 1M (red line), 2M (blue line), and 4M (green line) concentrations, using simple point charge (non-polarizable) force field MD simulations. For each concentration the plot is an average of 250000 trajectories. The simulation box setup was the same as with the polarizable FFMD, the SPC water model[2] was used together with Amber parameters for ions.[3,4]

Supplementary Table I. Simulated systems using polarizable force field MD.

Aqueous ionic solution	Concentration (mol/L)	Number of ions	Number of H_2O molecules	Cubic box side length in angstrom
Pure water	-	-	128	15.6404
Na_2SO_4	1	2	125	15.56
$MgCl_2$	1	3	191	17.9772
$MgCl_2$	2	5	133	16.099
$MgCl_2$	4	19	236	19.9629
NaCl	2	5	108	15.07
NaCl	4	8	104	15.00

Supplementary Table II. Molecular dipole moment, polarizabilites, first and second order hyperpolarizabilites of the gas phase water. Note that in our molecular frame of a water molecule, the x-axis points along the vector joining the two hydrogen atoms (H-H vector), y-axis the vector of the water bisector and the z-axis to the vector perpendicular to both water bisector and H-H vector.

**Property**	**H_2O Parameters**	**Units**
Dipole		Debye (atomic unit)
μ_y	1.93 (0.759)	
Polarizability		$Å^3$ (atomic unit)
α_{xx}	1.3725 (9.2713)	
α_{yy}	1.1580 (7.8224)	
α_{zz}	0.9127 (6.1653)	
First hyperpolarizability		$Å^5$ (atomic unit)
β_{yyy}	-0.5282 (-12.730)	
β_{xxy}	-0.6824 (-16.445)	
β_{zzy}	-0.2297 (-5.5365)	
Second hyperpolarizability		$Å^7$ (atomic unit)
γ_{xxxx}	3.1351 (269.8054)	
γ_{yyyy}	1.470 (126.5123)	
γ_{zzzz}	0.09407 (8.096200)	
γ_{xxyy}	2.1019 (180.8921)	
γ_{xxzz}	1.0817 (93.09720)	
γ_{yyxx}	2.1019 (180.8921)	
γ_{yyzz}	0.4124 (35.49410)	
γ_{zzxx}	1.0817 (93.09720)	
γ_{zzyy}	0.4124 (35.49410)	

References

1. Fukasawa, T. *et al.* Relation between Dielectric and Low-Frequency Raman Spectra of Hydrogen-Bond Liquids. *Phys. Rev. Lett.* **95**, 197802 (2005).
2. Berendsen, H. J. C., Grigera, J. R. & Straatsma, T. P. The missing term in effective pair potentials. *J. Phys. Chem.* **91**, 6269–6271 (1987).
3. Kashefolgheta, S. & Vila Verde, A. Developing force fields when experimental data is sparse: AMBER/GAFF-compatible parameters for inorganic and alkyl oxoanions. *Phys. Chem. Chem. Phys.* **19**, 20593–20607 (2017) and *Phys. Chem. Chem. Phys.* **20**, 28346-28347 (2018).
4. Cornell, W. D. *et al.* A Second Generation Force Field for the Simulation of Proteins, Nucleic Acids, and Organic Molecules. *J. Am. Chem. Soc.* **117**, 5179–5197 (1995).
